# Designing a tool for assessing injury severity parameters in order to develop a national Iranian ‎model for auditing preventable trauma-related death

**Published:** 2019-08

**Authors:** Homayoun Sadeghi-Bazargani, Hadi Jalilvand, Hassan Nouri Sari, Mohammad Meshkini, Pir-Hossein Kolivand, Alireza Ala, Seyyed Hossein Ojaghi Haghighi, Mohammad Asghari Jafarabadi, Farzad Rahmani, Reza Deljavan Anvari, Rouzbeh Rajaei Ghafouri, Yasin Sadeghi-Bazargani, Samad Shams Vahdati, Aysan Mohammad Namdar

**Affiliations:** ^*a*^Road Traffic Injury Research Center, Tabriz University of Medical Sciences, Tabriz, Iran.; ^*b*^Emergency Medicine Management Research Center, Rasoul-e-Akram Hospital, Iran University of Medical Sciences, Tehran, Iran.; ^*c*^Tehran Emergency Medical Service Center, Tehran, Iran.; ^*d*^Faculty of medicine, Tabriz University of Medical Sciences, Tabriz, Iran.

## Abstract

**Background:**

The medical definition of trauma is “any kind of intentional or unintentional, penetrating or ‎non-penetrating injury or wound caused by external causes”. Traumatic injury is a health threat ‎all over the world and the cause of 9% of worldwide mortality. Having a total of 258 trauma-‎scoring models indicates the versatile state of using trauma scores. Currently, there are no ‎national models for prediction of trauma-caused preventable death in Iran; This study, ‎conducted in 2018 and 2019, aimed to adapt a national model for Iran.‎

**Methods:**

Using appropriate keywords, the most commonly used scales for predicting death in traumatic ‎patients were searched from literature. These included ISS, GCS, TRISS, GAP, MGAP, PTS, ‎REMS, APACHE II, T-RTS, RTSc, NISS, MISS, and PHI. The potential trauma mortality ‎determinants and parameters were then identified and organized in a data collection form to be ‎measured through prospective examination of trauma cases after being assessed for its content ‎validity.‎

**Results:**

The final tool was comprised of three sections. The first section should be completed through ‎the first day of admission to an emergency department. It included the following items: pre-‎hospital health services, past medical history, medication use, Glasgow Coma Scale, vital signs, ‎routine laboratory tests in trauma, psychoactive and addictive drug history and alcohol misuse, ‎AIS index, PTS, trauma mechanism and external cause according to International Classification ‎of Diseases. The second section belonged to secondary evaluation 24 hours after the first ‎evaluation and included vital signs, GCS, AIS and admission decision. The third section was for ‎one-month follow-up and included the Glasgow Outcome Scale.‎

**Conclusions:**

The developed tool at three sections was found valid and applicable based on expert views and ‎identified feasible through its pilot administration.‎

**Keywords:**

Trauma score, Iran, Death, Predictors, National model

